# Analogous cellular contribution and healing mechanisms following digit amputation and phalangeal fracture in mice

**DOI:** 10.1002/reg2.51

**Published:** 2016-03-09

**Authors:** Lindsay A. Dawson, Jennifer Simkin, Michelle Sauque, Maegan Pela, Teresa Palkowski, Ken Muneoka

**Affiliations:** ^1^ Department of Cell and Molecular BiologyTulane UniversityNew OrleansLouisiana70118USA; ^2^ Department of BiologyUniversity of KentuckyLexingtonKentucky40506USA; ^3^ Department of OrthopedicsUniversity of Colorado DenverAuroraColorado80010USA; ^4^ Department of Veterinary Physiology and Pharmacology, College of Veterinary MedicineTexas A&M UniversityCollege StationTexas77843USA

**Keywords:** Digit, endochondral ossification, fracture, mouse, periosteum, regeneration

## Abstract

Regeneration of amputated structures is severely limited in humans and mice, with complete regeneration restricted to the distal portion of the terminal phalanx (P3). Here, we investigate the dynamic tissue repair response of the second phalangeal element (P2) post amputation in the adult mouse, and show that the repair response of the amputated bone is similar to the proximal P2 bone fragment in fracture healing. The regeneration‐incompetent P2 amputation response is characterized by periosteal endochondral ossification resulting in the deposition of new trabecular bone, corresponding to a significant increase in bone volume; however, this response is not associated with bone lengthening. We show that cells of the periosteum respond to amputation and fracture by contributing both chondrocytes and osteoblasts to the endochondral ossification response. Based on our studies, we suggest that the amputation response represents an attempt at regeneration that ultimately fails due to the lack of a distal organizing influence that is present in fracture healing.

## Introduction

Mammals, including humans and mice, lack the extensive regenerative capacity of urodele amphibians but are capable of regenerating the distal region of the terminal phalanx (P3) following amputation (Douglas 1972; Illingworth 1974; Borgens 1982). In mice, this response occurs in fetal, neonatal, and adult digits, and involves the formation of a blastema of undifferentiated and proliferating cells (Reginelli et al. 1995; Han et al. 2008; Fernando et al. 2011). The formation of a blastemal intermediate during regeneration distinguishes this as an epimorphic response that is distinct from tissue‐specific repair (Carlson 2007). Like the limb blastema of urodele amphibians (Kragl et al. 2009), there is evidence that cells of the mouse digit blastema come from multiple tissue types and that progenitor cells are lineage restricted (Lehoczky et al. 2011; Rinkevich et al. 2011; Takeo et al. 2013). The mouse digit regeneration model is unique in part because it occurs in a mammal, but also because it provides a way to explore amputation injury responses that are either regeneration‐competent or regeneration‐incompetent. As such, regeneration‐incompetent amputations at a more proximal digit or limb level have been used to test approaches aimed at enhancing the regenerative response (Masaki & Ide 2007; Agrawal et al. 2010, 2011a, 2011b; Yu et al. 2010, 2012; Ide 2012; Mu et al. 2013; Miura et al. 2015). Amputation at digit or limb levels proximal to the digit tip fail to regenerate; however, there is evidence that the healing response associated with the amputation injury can vary depending on the tissues present. For example, Schotté and Smith (1959) found that the bone healing response differed between mid‐diaphyseal amputations versus amputation levels approaching the epiphysis. For this reason, it is important to characterize the healing response of regeneration‐incompetent amputation injuries (Turner et al. 2010). In recent years, amputation through the middle of the second phalangeal element (P2) has been used as a regeneration‐incompetent model in neonatal and adult mice (Agrawal et al. 2010, 2011a, 2011b, 2012; Yu et al. 2012; Mu et al. 2013). In the neonate, we have shown that bone morphogenetic protein 2 (BMP‐2) induces a regenerative response in the amputated P2 bone that involves the formation of a transient blastema, the creation of an endochondral ossification center, and the patterned extension of the stump bone (Yu et al. 2012). Thus, there is proof of principle that regeneration can be induced from a regeneration‐incompetent amputation injury in mammals.

Mammalian bone has the potential to complete a successful regenerative response following traumatic injury, such as fractures, with an outcome that restores pre‐injury strength and morphology (Einhorn 2005; Al‐Aql et al. 2008; Shapiro 2008). In fracture healing, a blastema does not form; however, a response by the periosteum forms a proliferative cartilaginous callus that surrounds the injured bone and serves as a transient intermediate that organizes bone repair (Gerstenfeld et al. 2003; Tsuji et al. 2006; Schindeler et al. 2008; Colnot 2009; Wang et al. 2011). The periosteum is defined as the outer structure of the bone covering all non‐articular surfaces and is composed of two layers: a cell‐rich inner layer, termed the cambium layer, and a relatively cell‐poor outer layer, referred to as the fibrous layer (Ito et al. 2001; Dwek 2010). Bone fracture induces chondrogenic progenitor cells of the periosteum to proliferate and form the cartilaginous callus which then undergoes endochondral ossification to form new bone tissue (Ito et al. 2001; Tsuji et al. 2006; Colnot 2009; Dwek 2010). Both bone fragments of the fracture form a callus and the physical merger of the partnering calluses across the fracture plane serves to stabilize and repair the broken bone (Gerstenfeld et al. 2003). The endochondral ossification response of the callus initially differentiates into bone tissue with many trabeculae, termed woven bone, and this bony tissue is subsequently remodeled into cortical bone, thus restoring bone strength (Shapiro 2008). While it is clear that the regeneration of bone occurs during fracture healing but not following amputation, there are studies that describe the formation of a chondrogenic callus during bone healing following amputation (Schotté & Smith 1959, 1961; McKibbin 1978; Miura et al. 2015). Therefore, it seems likely that the initial response to amputation of the bone stump is an attempt at a regenerative response.

In adult mice, treatment of P2 amputations with cryptic peptides or metalloproteinases has been shown to recruit stem cells, induce ossification, and enhance soft tissue regeneration, but without elongation of skeletal structures (Agrawal et al. 2010, 2011a, 2011b, 2012; Mu et al. 2013). These studies fail to report any endogenous responses of untreated amputations, seemingly in conflict with previous studies of digit amputation injuries in rodents (Schotté & Smith 1959, 1961; McKibbin 1978). In this study, we compare the amputation response to the fracture healing response of the second phalanx in adult mice using quantitative micro‐computed tomography (μCT), histology, immunohistochemistry, and cell lineage tracking. We show that the early response to amputation is similar to the fracture healing response. Following digit amputation, a chondrogenic callus forms in lateral regions of the amputated bone and this response results in the deposition of new bone tissue that increases the diameter of the bone without increasing the proximal−distal length. Lineage tracking studies show that the chondrogenic callus is formed from the periosteum, and that the osteoblasts that form the new bone are also derived from the periosteum. μCT studies provide evidence that, at later stages, the newly formed bone is remodeled to a morphology that is similar to the freshly amputated bone; thus, it appears the digit stump is inert in its response to amputation. We also provide histological evidence that damaged tendon tissue in the ventral stump initially contracts but later elongates across the amputation surface and attaches to the dorsal region of the stump bone. These studies provide evidence that, following digit amputation, the tissues of the stump respond in a tissue‐specific manner in an attempt at regeneration that ultimately fails. We propose that there is value in understanding critical aspects of this response in devising therapeutic strategies for enhancing mammalian regeneration.

## Results

### Digit amputation

**Figure 1:**
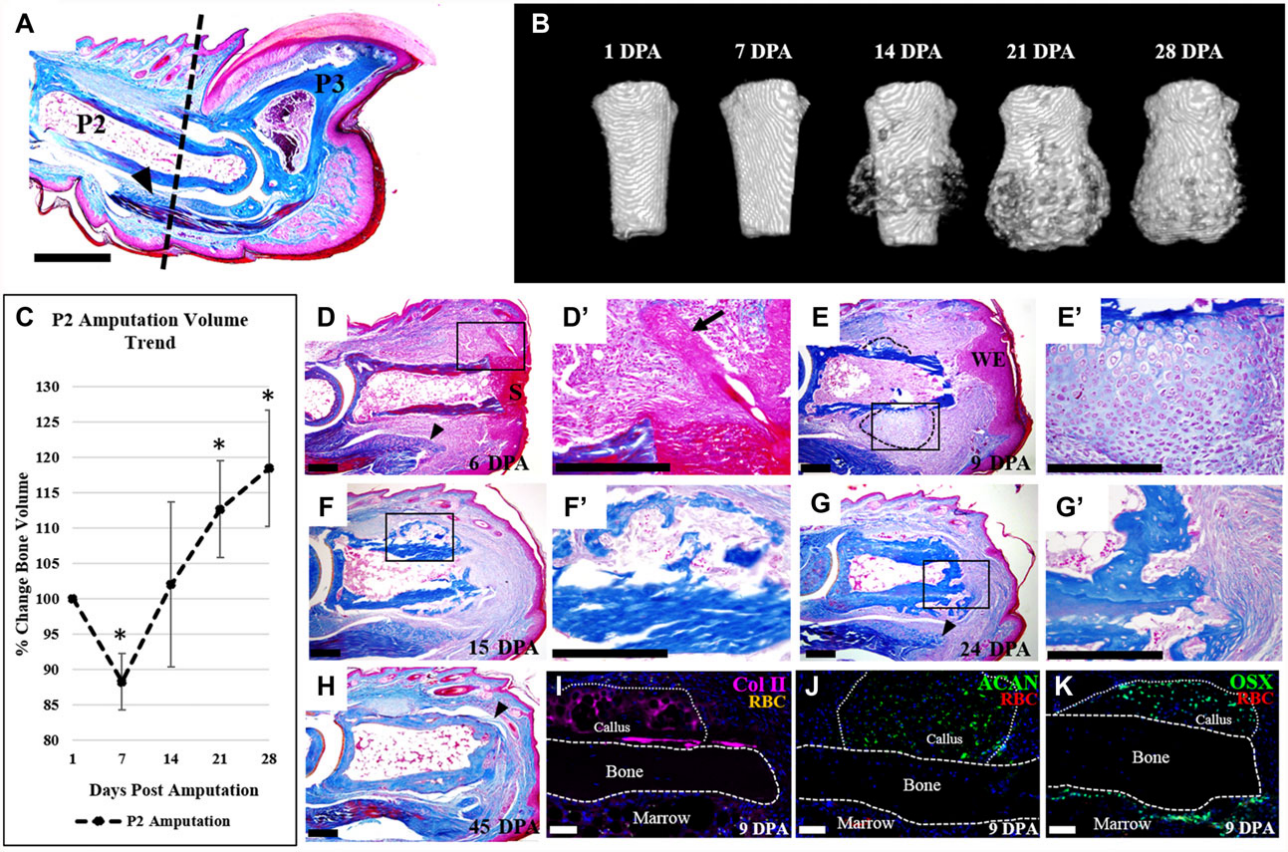
P2 amputation initiates local endochondral ossification response. (A) Mallory trichrome stained section of an adult mouse hind limb digit. Dashed line indicates the amputation level. (B) μCT reconstructed images show new bone formation initiated by 14 DPA, and continued bone formation at 21 and 28 DPA. (C) Bone volume measurements, normalized to 1 DPA, indicate bone degradation at 7 DPA, resulting in an average bone loss of 12%, followed by a bone growth phase, resulting in an 18% overall increase in bone volume by 28 DPA (*t* test, ±SEM, *P < 0.005). (D)−(H) Histological sections of digits stained with Mallory trichrome stain. (D), (D′) At 6 DPA wound closure has not completed and wound retraction is evident. (E), (E′) Wound closure is completed by 9 DPA, and cartilaginous growth is evident on the lateral portions of the bone (outlined). (F), (F′) Replacement of the cartilaginous callus with woven bone and marrow cells is apparent by 15 DPA. (G), (G′) By 24 DPA, a bone plug has formed at the distal portion of the stump. (H) Bone remodeling and tendon reattachment to the bone (arrowhead) is evident by 45 DPA. (I)−(K) Immunostaining of 9 DPA samples, counterstained with DAPI. (I) Col II immunostaining indicates cartilaginous tissue along the periosteal surface. (J) Immunostaining for aggrecan (ACAN) confirming cartilaginous tissue is present. (K) Immunostaining for osterix (Osx) reveals osteoblasts localized to the periosteal callus and the endosteum. (A), (D)−(K) Dorsal surface is top, distal to the right. (B) Top is proximal, bottom is distal. S, scab; WE, wound epidermis; RBC, red blood cells. Scale bars: (A) 500 μm; (D)−(H) 200 μm; (I)−(K) 50 μm.

The amputation of phalangeal bones has been used as a model of epimorphic mammalian regeneration: amputation of P3 results in blastema formation and a successful regenerative response, whereas amputation at more proximal levels undergoes wound healing without skeletal regeneration. Amputation of P2 has emerged as a non‐regenerating model to test treatments designed to enhance the regenerative response. Amputation transecting the digit at the P2 level results in scar formation, characterized by the formation of fibrous tissue capping the bone stump and a lack of distal bone growth (Turner et al. 2010; Yu et al. 2010, 2012; Agrawal et al. 2011a, 2011b, 2012; Mu et al. 2013). The detailed anatomy of the mouse digit is nicely described by Wong et al. (2006) and summarized here (Fig. 1A). The P2 bone is a dominant, centrally located structure that is surrounded by interstitial connective tissue, a ventrally positioned deep digital tendon with associated fibrocartilage (Fig. 1A, arrowhead), lateral nerves that run the length of the bone (not shown), and a dorsally positioned claw ligament. The digit skin differs dramatically between the dorsal epidermis, which is rich with hair follicles, and the ventral plantar epidermis that lacks hair. The P2 bone contains a marrow region with a sparse cell population and is not as highly vascularized by comparison to the P3 marrow.

To better characterize the P2 amputation response, we use μCT imaging to document anatomical and volume changes of the P2 bone (Fig. 1B, C, *n* = 12). μCT imaging indicates that during the first 7 days post amputation (DPA) there are few obvious anatomical changes to the bone stump, but by 14 DPA there is an initial circumferential deposition of new bone. This new bone deposition continues and results in a significant increase in stump bone volume at 21 and 28 DPA compared to the bone volume at the time of amputation (Fig. 1C, *P* < 0.005). We note that, while we do not observe gross anatomical changes, there is a significant decrease in stump bone volume at 7 DPA (Fig. 1C, *P* < 0.005). This temporal pattern of stump bone volume changes is similar to that observed during the regenerative response following P3 amputation (Fernando et al. 2011); however, the P2 response does not result in an increase in the proximal−distal length of the P2 bone. While we do not find evidence for overt regeneration, the anatomical changes of the P2 stump bone indicate that the response to amputation injury is not static but is, in fact, quite dynamic. Since a number of recent investigative studies aimed at enhancing the mammalian regenerative response use the P2 digit amputation wound, we believe that an in‐depth analysis of this dynamic response will be valuable.

We carried out a detailed histological analysis of amputated digits at various time points during the healing response. At 6 DPA, wound closure is not complete and a scab still covers the distal bone stump (Fig. 1D). The positioning and orientation of hair follicles in close proximity to the P2 stump bone are suggestive of considerable wound contraction (Fig. 1D′, arrow). There is also evidence of edema of the soft tissue surrounding the stump and there is an increased cell density within the P2 marrow cavity. We note histological evidence of bone thinning that is in agreement with bone volume measurements from μCT scans. The deep digital flexor tendon is prominent in the ventral−proximal region and appears to have contracted away from the amputation wound (Fig. 1D, arrowhead). At 9 DPA, wound closure is complete and the wound epidermis is thickened in some regions of the stump (Fig. 1E). The distal region of the bone marrow cavity is open to the amputation wound and the cavity itself is highly cellular. At this stage, a prominent chondrogenic callus has formed circumferentially in the lateral regions of the P2 stump (Fig. 1E, outlined; 1E′). By 15 DPA, there is histological evidence of circumferential ossification of the stump bone (Fig. 1F, F′) that is consistent with μCT images. Because the circumferential ossification response is associated with a chondrogenic pre‐condition, the histological evidence points to the activation of an endochondral ossification process that results in the increase in stump bone volume. At 15 DPA, the distal stump is still open and the marrow region is contiguous with the soft tissues of the amputation wound (Fig. 1F). At 24 DPA, circumferential ossification of the stump is apparent and a bone plug caps the stump, distally sealing the bone marrow cavity from the wound site (Fig. 1G, G′). We note that, unlike the circumferential bone growth, we observe no chondrogenic template at the distal stump at any time point analyzed, suggesting that the bone plug forms by direct ossification. At 24 DPA the cellularity of the bone marrow region appears to have returned to a pre‐amputation state. In the ventral soft tissue, the deep digital flexor tendon is extended distally toward the amputation wound (Fig. 1G, arrowhead). By 45 DPA, woven bone of the callus has been remodeled and the resulting bone more closely resembles the original bone diameter (Fig. 1H). Thus, the formation of the chondrogenic callus and subsequent appearance of circumferential woven bone are transient responses of the P2 bone to amputation injury. Finally, we note that the deep digital flexor tendon is prominent and a number of fibers can be traced across the digit stump, forming insertion points on the dorsal aspect of the stump bone (Fig. 1H, arrowhead). While this response is anatomically abnormal, it is suggestive of an attempted repair response of a tissue that is generally accepted to be non‐regenerative (Fig. 1H).

One remarkable characteristic of the amputation response is the formation of a chondrogenic callus that precedes the formation of new bone around the circumference of the P2 stump. This response initiates during the second week after amputation and is associated with the periosteum of the bone stump. We used immunostaining of samples at 9 DPA with two different markers for chondrocytes, type II collagen (Col II) and aggrecan (ACAN), to verify whether the circumferential callus was indeed chondrogenic. Immunostaining for Col II and ACAN identified cells within the callus, whereas we did not observe any chondrocytes associated with the endosteum, within the bone marrow cavity itself, or distal to the bone stump (Fig. 1I, J). Using osterix (OSX) as a marker for osteoblasts, we also found immunostained cells within the callus and along the endosteal layer but absent from the periosteum (Fig. 1K), indicating that osteogenesis was occurring within the chondrogenic callus as well as within the bone marrow. These data are consistent with the conclusion that amputation stimulates enhanced ossification by the bone stump: an endochondral ossification response by the periosteum and a direct ossification response by the endosteum. The end result is a significant increase in stump bone volume without any obvious lengthening of the stump bone, consistent with our μCT analysis.

### Similarities with fracture healing

**Figure 2:**
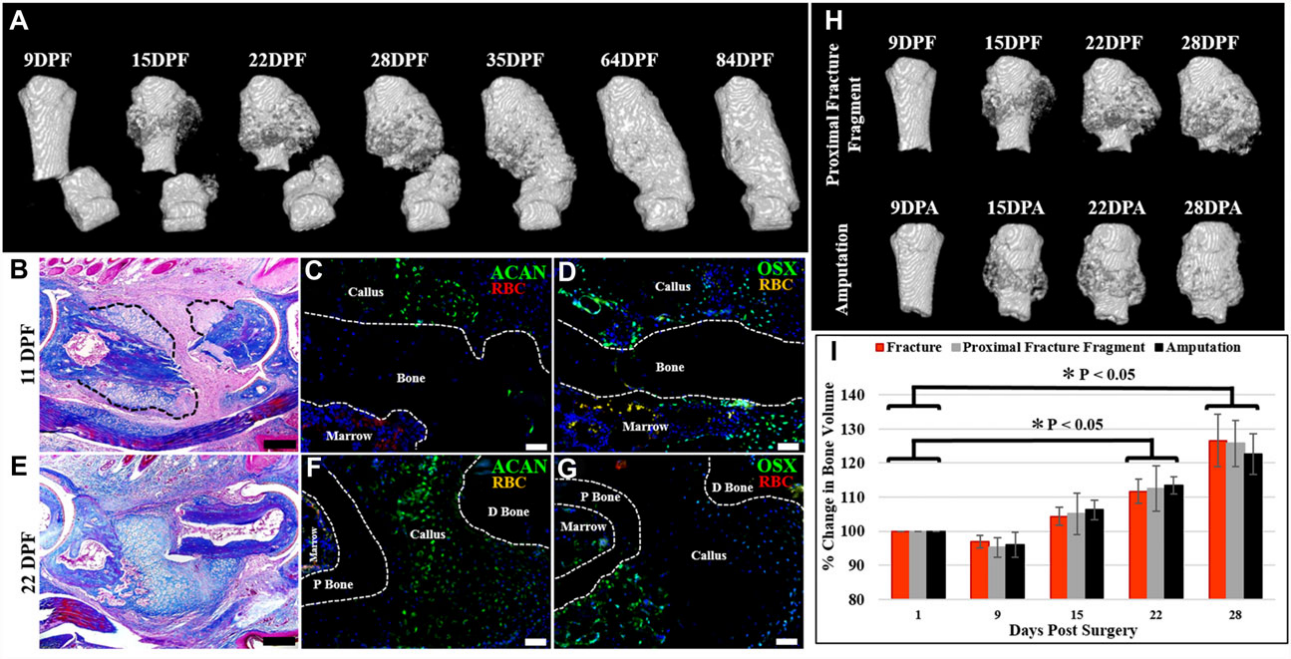
The repair response following amputation is similar to fracture healing. (A), (I) μCT reconstructed images of the fractured P2 digit show periosteal bone formation evident by 15 DPF, with progressive bone growth and eventual merging of the proximal and distal bone fragments by 35 DPF, coinciding with a significant increase in bone volume when normalized to 1 DPF (*t* test, ±SEM, *P < 0.05). (B)−(D) 11 DPF serial sectioned sample. (B) Mallory trichrome stained section showing cartilaginous growth on the proximal and distal bone segments (outlined). (C) Immunostaining for ACAN confirming cartilaginous cells associated with the periosteum. (D) OSX immunostaining showing osteoblasts localized to the callus and the endosteum/marrow. (E)−(G) 22 DPF serial sectioned sample. (E) Mallory trichrome stained section showing new bone and marrow formation on the proximal bone fragment and cartilaginous bridging of the fracture gap. (F) Immunostaining for ACAN showing cartilage spanning the fracture gap. (G) Immunostaining for OSX showing osteoblasts localized to the proximal bone fragment. (C), (D), (F), (G) Samples counterstained with DAPI. (H) Comparative analysis of μCT reconstructed images showing that the proximal fracture bone fragment is morphologically similar to the amputation stump, with ossification apparent by 15 DPF/DPA, and continued bone formation at 22 and 28 DPF/DPA. (I) Bone volume measurements indicate no statistical significance between the three experimental groups when normalized to the starting bone volume (*t* test, ±SEM, *P* < 0.05). (B)−(G) Dorsal surface is top, distal to the right. (A), (H) Top is proximal, bottom is distal. P bone, proximal bone fragment; D bone, distal bone fragment; RBC, red blood cells. Scale bars: (B), (E) 200 μm; (C), (D), (F), (G) 50 μm.

The formation of a chondrogenic callus following digit amputation is reminiscent of the response of long bones during fracture repair (Gerstenfeld et al. 2003; Shapiro 2008; Colnot 2009). A recent study not only highlighted morphological similarities between the cartilaginous callus formed in response to amputation or fracture of the neonate mouse forelimb, but also demonstrated a shared nerve independence of cartilage formation following denervation (Miura et al. 2015). Despite evidence suggesting that amputation injury is uniquely different from fracture injury in long bones (Mckibbin 1978), our observations suggest that the phalangeal response may be quite similar. For this reason, we carried out a series of studies comparing the P2 amputation response to P2 fracture healing. The P2 bone of the mouse was fractured by simple transection using micro‐scissors and no attempt was made to stabilize the fracture. μCT analyses of the response are shown in Figure 2A. The alignment of the proximal and distal bone fragments was highly variable, although the repaired fractures resulted in a contiguous P2 bone which seamlessly merged the two initial bone fragments at later stages. Thus, like long bone fracture healing, the phalangeal fracture response results in the bridging of the proximal and distal bone fragments. Based on μCT analyses, new bone formation is not observed at 9 days post fracture (DPF), coinciding with a small yet significant decline in bone volume, but is apparent by 15 DPF and onward (Fig. 2A, I, *n* = 4; *P* < 0.05). Like our amputation response, there is initial circumferential ossification, but in fracture healing the ossification response moves toward the fracture gap and appears to merge with a similar ossification response associated with the distal bone fragment (Fig. 2A). For our comparative analysis to amputation healing, we focused on the response of the proximal bone fragment throughout the initial 28 days during which there was no indication of osteogenic bridging across the fracture wound (Fig. 2B−I). Histological analysis at 11 DPF shows extensive circumferential callus formation with clear evidence of chondrogenesis (Fig. 2B, outlined). Immunostaining for ACAN verifies that the callus is chondrogenic at this stage (Fig. 2C), and immunostaining for OSX indicates that osteogenesis is occurring within the callus (Fig. 2D). Like our amputation studies, we do not observe chondrogenesis within the fracture bone marrow but we do find cells positive for OSX. By 22 DPF, the region of callus formation spans the distal gap between the proximal and distal bone fragments, indicative of cartilaginous formation prior to osteogenic bridging (Fig. 2E). Immunostaining for ACAN confirms that the callus is chondrogenic (Fig. 2F) and immunostaining for OSX (Fig. 2G) indicates that osteogenesis is occurring within the callus, predominantly in areas not immunopositive for chondrogenesis. Indeed, comparative μCT analysis of the proximal fracture bone fragment prior to bridging of the fracture gap shows the morphological and volume similarities to the amputation response (*n* = 4), with new circumferential bone formation and a corresponding increase in bone volume apparent by 22 days post injury, as well as no statistical significance between the injury types (Fig. 2H, I; *P* < 0.05). Moreover, comparative bone length analysis of the proximal fracture fragment and the amputation injury showed no increase in bone length by 28 days post injury, and no statistical difference between the two injuries at any time point (Fig. S1; *N* = 4; *P* < 0.05). All injury models show an increase in bone volume when normalized to the starting volume, with statistical significance apparent by 22 and 28 days post injury (Fig. 2I; *P* < 0.05).

These studies indicate that the early circumferential endochondral ossification response associated with fracture healing is similar to the amputation response. However, unlike digit amputation, the later stages of fracture healing extend the callus distally, resulting in newly regenerated bone that bridges the fracture gap. Since the primary goal of regeneration from an amputation injury is to form newly patterned bone distal to the amputation, it seems likely that understanding the mechanistic difference between amputation and fracture healing will shed light on approaches to enhance regeneration in mammals.

### Cell contribution

**Figure 3:**
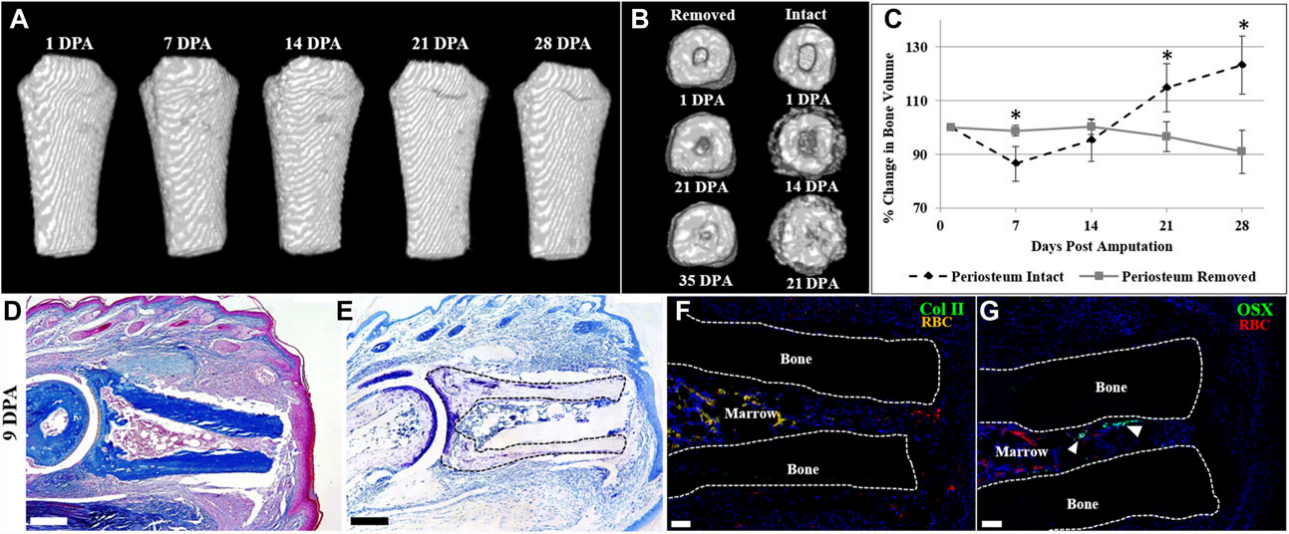
Periosteum removal inhibits cartilaginous callus formation after amputation. (A) μCT reconstructed images of the periosteum‐removed digit showing no discernable morphological change and no significant change in bone volume when normalized to 1 DPA (*t* test, ±SEM, *P* < 0.05). (B) Distal end view of μCT reconstructed images of periosteum‐removed digits showing marrow closure by 35 DPA versus marrow closure by 21 DPA in digits with an intact periosteum. (C) Bone volume measurements over 28 days, normalized to 1 DPA, showing no significant volume change in periosteum‐removed digits. This pattern is significantly different from periosteum‐intact digits (*t* test, ±SEM, *P < 0.05). (D)−(G) Serial sections of a representative periosteum‐removed digit at 9 DPA. (D) Mallory trichrome staining illustrating no circumferential callus formation on the amputated bone. (E) Toluidine blue staining showing cartilage present in the articular region and no cartilaginous staining on the stump bone surface. (F) Col II immunostaining confirming the absence of cartilage formation on the periosteal surface. (G) OSX immunostaining showing no osteoblasts on the periosteal surface and several immunopositive cells in the endosteum/marrow space (arrowheads). (F), (G) Samples counterstained with DAPI. (A) Top is proximal, bottom is distal. (D)−(G) Dorsal surface is top, distal to the right. RBC, red blood cells. Scale bars: (D), (E) 200 μm; (F), (G) 50 μm.

To further investigate the relationship between fracture healing and the amputation response of the mouse digit, we carried out experiments on P2 amputated bone to study chondrogenic callus formation. Previous long bone fracture healing studies demonstrated that the cartilaginous component of the fracture callus is derived from the periosteum (Colnot 2009). We first carried out P2 amputation studies in which the periosteum was mechanically removed at the time of digit amputation. Sham‐operated control digits were analyzed in parallel. μCT studies of periosteum‐removed digit amputations showed that, in the absence of the periosteum, there is no observable circumferential anatomical change in bone morphology and that bone volume remained unchanged for a 28‐day period (Fig. 3A, C;*N* = 4; *P* < 0.05). We observed new bone formation at the distal bone stump, plugging the marrow space from the surrounding tissues, in both sham and periosteum‐removed samples (Fig. 3B; *N* = 4; see Fig. 1G, G′). In sham‐operated controls, plugging was nearly complete at 21 DPA, while periosteum‐removed samples showed a relatively slower organization of distal closure, with plugging nearing completion by 35 DPA (Fig. 3B; *N* = 4). Sham‐operated controls displayed a bone volume response that was identical to simple digit amputation as shown in Figure 1, with the increase in bone volume differing significantly from periosteum‐removed samples at 21 and 28 DPA (Fig. 3C; *P* < 0.05). The unchanging bone volume following amputation of periosteum‐removed digits is consistent with the conclusion that the periosteum is involved in the deposition of circumferential bone that occurs in response to amputation. Histological studies at 9 DPA showed that the periosteum‐removed bone stump failed to form a circumferential callus but the epidermal healing response and other tissues of the digit stump appeared normal (Fig. 3D). Toluidine blue staining of the periosteum‐removed digit showed cartilaginous stained cells within the P1/P2 joint region but failed to identify any cartilaginous tissues typically associated with the P2 stump bone (Fig. 3E, outlined). Immunostaining for Col II and OSX verified the absence of chondrocytes and osteoblasts adjacent to the periosteal surface associated with the amputation response of the periosteum‐intact digits (Fig. 3F, G). We observed OSX‐positive osteoblasts in the endosteal marrow region of periosteum‐removed digits (arrowheads), consistent with our μCT findings of bone formation plugging the stump (Fig. 3B, G). These periosteum removal studies suggest that the periosteum plays a key role in cartilage callus formation in response to digit amputation, a conclusion supported by similar studies in fracture healing (Colnot 2009).

**Figure 4:**
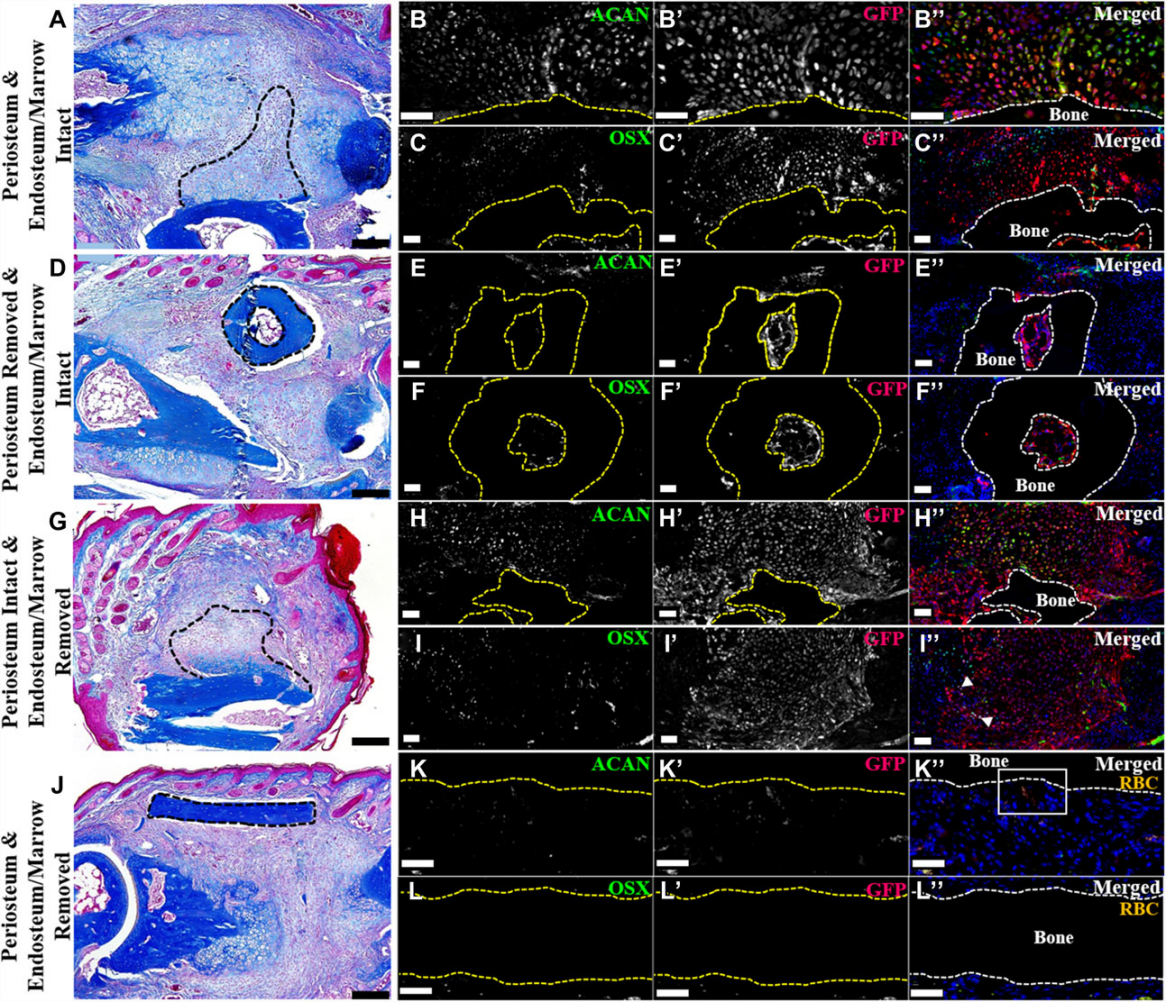
P2 periosteum and endosteum/marrow contribute to wound repair (dorsal surface is top, distal to the right). (A)−(C′′) Serial sections of an intact GFP‐labeled P2 bone grafted into a NOD‐SCID fractured P2 digit, 11 DPF. Representative sample shown. *N* = 4. (A) Mallory trichrome staining showing callus formation on the grafted bone (outlined). (B)−(B′′) Double immunostaining for ACAN and GFP illustrating cartilage derived from the bone graft (outlined). (C)−(C′′) Double immunostaining for OSX and GFP showing graft‐derived osteoblasts within the periosteal callus and the endosteal/marrow space (graft outlined). Immunohistochemical stained samples counterstained with DAPI. (D)−(F′′) Serial sections of a periosteum‐removed and endosteum/marrow‐intact GFP‐labeled P2 bone grafted into a NOD‐SCID fractured P2 digit, 11 DPF. Representative sample shown. *N* = 4. (D) Mallory trichrome staining showing no periosteal callus formation on the grafted bone (outlined). (E)−(E′′) Double immunostaining for ACAN and GFP revealing no graft‐derived chondrocytes present on the periosteal surface or within the marrow cavity (graft outlined). (F)−(F′′) Double immunostaining for OSX and GFP showing double‐labeled osteoblasts present within the graft marrow space (outlined). Immunohistochemical stained samples counterstained with DAPI. (G)−(I′′) Serial sections of periosteum‐intact and endosteum/marrow‐removed GFP‐labeled P2 bone grafted into a NOD‐SCID fractured P2 digit, 11 DPF. Representative sample shown. *N* = 4. (G) Mallory trichrome staining showing graft periosteal callus formation (outlined). (H)−(H′′) Double immunostaining for ACAN and GFP showing the graft‐derived cartilaginous callus (grafted bone outlined). (I)−(I′′) OSX and GFP double immunostaining showing graft‐derived osteoblasts (arrowheads). Immunohistochemical stained samples counterstained with DAPI. (J)−(L′′) Serial sections of a periosteum and endosteum/marrow‐removed GFP‐labeled P2 bone grafted into a NOD‐SCID fractured P2 digit, 11 DPF. Representative sample shown. *N* = 4. (J) Mallory trichrome staining showing no callus formation on the grafted bone (outlined). (K)−(K′′) Double immunostaining for ACAN and GFP indicating no graft‐derived chondrocytes present (grafted bone outline). (L)−(L′′) Double labeled osteoblasts were not detected by OSX and GFP double immunostaining (grafted bone outlined). (K)−(L′′) Signal indicates red blood cells (RBC). Immunohistochemical stained samples counterstained with DAPI. Scale bars: (A), (D), (G), (J) 200 μm; all immunostaining 50 μm.

To further probe the cellular origin of the amputation callus, we carried out studies in which green fluorescent protein (GFP) labeled amputated P2 bone was engrafted into a P2 fracture site in unlabeled NOD‐SCID mice. Using this engraftment approach, we analyzed callus formation at 11 days post engraftment when chondrogenesis is prominent of (1) intact amputated bone, (2) amputated bone with the periosteum mechanically removed, (3) amputated bone with the endosteum/marrow mechanically removed, and (4) amputated bone with periosteum and endosteum/marrow mechanically removed (*N* = 4 for each engraftment approach). In preliminary studies, we observed no residual transgenic GFP fluorescence after tissue processing (Fig. S2A); consequently, in order to visualize GFP‐positive cells we performed immunostaining against GFP. Histological staining of bone grafts with intact periosteum and endosteum shows a large area of cartilage directly adjacent to the periosteal surface but absent in the engrafted marrow region (Fig. 4A, outlined). We performed immunohistochemical analysis of adjacent sections and found that the callus was GFP‐labeled and that many of the cells co‐expressed the chondrocyte marker ACAN, indicating that chondrogenic cells within the callus were derived from grafted bone tissue (Fig. 4B−B′′). We note that not all GFP‐positive cells tested immunopositive for ACAN, suggestive of a heterogeneous population of cells stemming from the labeled bone graft (Fig. 4B−B′′). Analysis of serial sections shows that cells in the periosteal callus and also associated with the endosteum within the bone marrow co‐express GFP and OSX, indicating that osteogenic cells of the callus and endosteum were derived from the grafted bone tissue (Fig. 4C−C′′). These data are consistent with the conclusion that chondroprogenitor and osteoprogenitor cells of the periosteum participate in callus formation following P2 digit amputation.

Histological staining of cell labeling studies using P2 bone stump grafts in which the periosteum is mechanically removed show that callus formation is largely absent (Fig. 4D). Immunohistochemical analysis confirms our findings, with few GFP/ACAN or GFP/OSX double‐labeled cells observed adjacent to the graft periosteum, whereas GFP‐labeled osteoblasts are abundant within the graft marrow compartment (Fig. 4E−F′′). Analysis of P2 bone stumps in which the endosteum and marrow are mechanically removed but the periosteum is intact indicates robust callus formation (Fig. 4G) with an abundance of GFP/ACAN double‐labeled cells (Fig. 4H−H′′). In these grafts, there is a paucity of GFP‐labeled cells in the marrow region and few GFP/OSX double‐labeled cells (Fig. 4H−I′′). Histological staining of labeled P2 bone stumps with periosteum and endosteum mechanically removed do not show chondrogenesis or new bone growth adjacent to the graft, suggesting a lack of cortical bone contribution to the healing P2 bone (Fig. 4J). Immunohistochemical analysis of serial sections validates our histological observation, showing no GFP/ACAN or GFP/OSX double‐labeled cells and only the presence of autofluorescent red blood cells indicative of vascular invasion (Fig. 4K−L′′; for K′′ inset, refer to Fig. S2B). Overall, these results parallel similar studies of the cellular contribution to the fracture healing response (Colnot 2009) and support the conclusion that, following P2 digit amputation, the bone stump undergoes an initial chondrogenic response by the periosteum that is followed by an ossification response and ultimately leads to an increase in stump bone volume. While digit amputation does not result in the lengthening of the P2 bone (i.e., regeneration), the stump tissue reacts to the injury by producing new bone tissue (i.e., tissue regeneration) that is organized circumferentially around the stump. These periosteal cells therefore represent a target cell population for therapies aimed at enhancing the regenerative response following amputation.

## Discussion

The mouse digit is an established model to study epimorphic regeneration in mammals (Simkin et al. 2015a). It is one of a few mammalian models for epimorphic regeneration and, because the regenerative response is level‐dependent, it represents an important translational paradigm in which regenerative therapies can be tested. We have used neonatal digit amputations to provide definitive proof of concept evidence that enhanced regeneration is possible, and that the response is level‐dependent (Yu et al. 2010, 2012). The evidence shows that the positional information matrix necessary for a regenerative response remains intact in tissues that are known to be regeneration‐incompetent (Yu et al. 2012; Simkin et al. 2015a). Studying differences between regeneration‐competent and regeneration‐incompetent amputation injuries in a mammal makes this model particularly attractive for uncovering therapeutic approaches in regenerative medicine. In addition, the maturity of molecular genetic manipulation in the mouse (Lehoczky et al. 2011; Rinkevich et al. 2011; Takeo et al. 2013) and the routine use of non‐invasive imaging techniques, such as μCT (Fernando et al. 2011; Yu et al. 2012; Sammarco et al. 2014; Simkin et al. 2015b), provide both qualitative and quantitative approaches that are not available in other regeneration models. While there are a number of studies detailing endogenous regeneration of adult digit tips (Fernando et al. 2011; Rinkevich et al. 2011; Takeo et al. 2013; Sammarco et al. 2014; Simkin et al. 2015b), less attention has been given to the healing response associated with a non‐regenerative amputation wound. Since any digit or limb amputation at a level proximal to the digit tip fails to regenerate, the selection of an amputation level to focus on is quite arbitrary. A number of recent studies have used amputation at the level of the second phalangeal element (P2) as a model of regenerative failure to investigate strategies aimed at enhancing/inducing regenerative responses. Such studies have identified extracellular matrix degradation products (e.g., cryptic peptides) and metalloproteinase production as potential stimulators of a regenerative response (Agrawal et al. 2011a, 2011b; Mu et al. 2013). In the current investigation, we have focused on this P2 amputation model and show that the amputation injury response is not inert but quite dynamic with bone tissue displaying a novel biphasic (degradation/new bone deposition) response that parallels the fracture healing response of the P2 element. Additionally, tendon regrowth and reattachment to the bone occurs in these digits. These findings emphasize the need for careful characterization and quantitation of the healing response when probing strategies in regenerative medicine.

Our study adds to a handful of previous studies investigating the effect of amputation on the mouse digit that yield varying results. Consistent with our findings, Schotté and Smith (1959) examined forelimb digits of outbred juvenile (4−6 weeks old) Swiss mice and reported callus formation and osteogenesis following amputation through the P2 diaphysis. In contrast, the formation of a lateral callus does not occur in mid‐level P2 amputations of the hind limb digit in juvenile (6−8 weeks old) C57/BL6 mice, but lateral osteogenesis can be induced by treatment with a cryptic peptide (Turner et al. 2010; Agrawal et al. 2011b). The histological similarity between cryptic peptide induced ossification in the C57/BL6 digit amputation and the endogenous osteogenic response associated with callus formation observed in outbred mice is striking. While callus formation does occur during fracture healing or digit amputation in adult C57/BL6 mice (Wang et al. 2006), strain‐specific differences in regenerative ability following digit amputation in neonates have been noted (Chadwick et al. 2007; Lehoczky et al. 2011). A lateral ossification response does not occur following P2 amputation in neonatal outbred mice (Yu et al. 2012); however, a lateral callus does form following neonatal limb amputation (Ide 2012; Miura et al. 2015). Thus, the absence of a callus formation response following P2 digit amputation in juvenile mice may represent a strain‐ and/or development‐specific defect in C57/BL6 juveniles that appears to be rescuable with cryptic peptide treatment. Whether or not cryptic peptide release is causally linked to endogenous callus formation requires further study.

The best understood model of adult bone regeneration is the fracture healing response. Successful fracture healing is dependent upon adequate bone reduction, sufficient vascular supply, the extent of injury to the surrounding soft tissues, and immobilization at the fracture site (Nunamaker et al. 1985). Of the nearly 8 million fractures that occur yearly in the USA, an estimated 10% fail to heal properly, most notably leading to nonunion of the bone segments, creating an important obstacle for orthopedic care (Sathyendra & Darowish 2013). Successful fracture healing involves the formation of a circumferential callus that surrounds the bone in proximity to the fracture. Bone tissue both proximal and distal to the fracture undergoes a similar response that involves the proliferation of cells of the periosteum to form a cartilaginous callus that undergoes endochondral ossification, followed by the formation of woven bone (Le et al. 2001; Thompson et al. 2001; Gerstenfeld et al. 2003; Colnot 2009; Histing et al. 2010). In successful healing, the proximal and distal callus bridges the fracture gap and, during later stages, the woven bone is remodeled into lamellar bone (Shapiro 2008). Our studies show that after simple amputation of the P2 bone the initial response of the stump bone is very similar to the proximal half of a fracture healing response. This includes (1) the participation of periosteal cells in the formation of a cartilaginous callus, (2) the differentiation into woven bone, and (3) a late remodeling event to form lamellar bone. Our studies indicate that the P2 periosteum contributes both chondrocytes and osteoblasts to the circumferential endochondral ossification response, while the endosteal/marrow‐derived cells differentiate directly to bone. Notably, in the absence of the periosteum, the characteristic early decrease in bone volume following amputation is inhibited as is the increase in bone volume observed at later time points. This suggests that the endochondral ossification response of the periosteum is triggered by an early bone degradation event and supports the general model that tissue histolysis is required for the release and/or activation of progenitor cells necessary for a regenerative response (Simkin et al. 2015b).

Both amputation and fracture healing responses are associated with an increase in bone volume. One clear difference between fracture healing and amputation, however, is that the amputation response does not result in new bone distal to the amputation plane; thus skeletal elongation does not occur. A simple conclusion from these observations is that the endochondral calluses of the partnering halves of the fractured bone must be interacting to successfully bridge the fracture gap. Since BMP signaling is known to be required for successful fracture healing (Tsuji et al. 2006; Wang et al. 2011) and BMP‐2 signaling can induce a distal endochondral ossification response (Yu et al. 2010, 2012), the data suggest that one action of the partnering halves of a fracture is to provide an additional source of a BMP signal. Furthermore, the endochondral ossification response of BMP‐2‐induced digit regeneration is indicative of a periosteal contribution to the regenerate. Studies on the induction of an endochondral regenerative response induced by BMP‐2 show that there is a direct influence on cell proliferation (Yu et al. 2012), as well as an indirect effect on cell recruitment involving endothelial cells and stimulation of the SDF‐1/CXCR4 signaling pathway (Lee et al. 2013). SDF‐1/CXCR4 signaling is also known to play a critical role in cell recruitment associated with successful fracture healing (Kitaori et al. 2009). These similarities provide further support for the conclusion that the response of P2 to amputation injury is similar to fracture injury.

Epimorphic regeneration has been defined and redefined over the years, and is now generally accepted to be a response that involves the formation of a blastema (Morgan 1901; Carlson 2007). During the regeneration of the mouse digit tip, the blastema is an accumulation of undifferentiated cells at the amputation site, and there is evidence that the blastema itself is composed of a number of subpopulations of lineage restricted cell types derived from different tissues of the stump (Lehoczky et al. 2011; Rinkevich et al. 2011; Takeo et al. 2013). Since tissue‐specific regeneration is generally not considered an epimorphic response, that is, it occurs in the absence of a blastema, the conclusion that lineage restricted cells contribute to the blastema (Kragl et al. 2009; Lehoczky et al. 2011) blurs the distinction between these two types of response. In this light, it seems evident that the epimorphic regenerative response in mice, and perhaps in other organisms, evolved to incorporate cellular mechanisms critical for tissue‐specific homeostatic replacement and/or injury repair, and that it will be important to probe the relationship between these two historically distinct categories of regenerative responses. For example, there are recent studies that have explored the fracture healing response in animals that have a high capacity for regeneration (Hutchison et al. 2007), and the evidence suggests that grafted blastema cells have the ability to participate in a tissue level repair response to bridge a critical size fracture gap (Satoh et al. 2010). Such studies point to the adaptive plasticity of blastema cells to conform to a tissue level repair response. Thus, the blastemal response to injury does not appear to be restricted by the nature of the injury, that is, amputation versus fracture. We suggest that a new paradigm in mammalian regeneration is to understand how to reinvent a blastema from subpopulations of tissue‐specific, lineage restricted progenitor cells that have the ability to undergo individual tissue level repair.

## Materials and methods

### Animals, P2 amputation, periosteum removal, and fracture

Adult 8‐week‐old, female CD‐1 mice were purchased from Harlan Laboratories (Indianapolis, IN), and adult female 8‐week‐old actin−enhanced GFP (actin‐EGFP) and NOD‐SCID mice were purchased from the Jackson Laboratory (Bar Harbor, ME). Anesthetization of mice was performed using isoflurane, with an initial dose of 3% isoflurane and maintained at 2% over the duration of the surgery. P2 amputations on hind limb digits two and four were performed using a scalpel to sever the digit at approximately the second ventral fat pad indent, mid‐way through the P2 bone (Fig. 1A). Digits in which the periosteum was mechanically removed were amputated at the P2 level followed by removal of the periosteum at the time of amputation. To remove the periosteum, P2 digits were amputated, the skin and surrounding soft tissues were pulled back, exposing the bone, and the periosteum was stripped off the entire bone surface using a scalpel. The soft tissues and skin were pulled forward to their original location, and the distal wound was closed using Dermabond (Ethicon, Somerville, NJ). Control sham digits were treated the same not including periosteum scraping. P2 open fractures were carried out by an initial micro‐scissor incision, creating a small hole thus exposing the P2 bone, followed by fracture of the bone through the use of small surgery scissors. The open wound was closed using Dermabond (Ethicon). The P2 bone was fractured at approximately the second ventral fat pad indent, coinciding with the P2 amputation level. All animal use and techniques were in compliance with the standard operating procedures of Tulane University's Institutional Animal Care and Use Committee.

### EGFP‐labeled P2 bone grafts

Actin‐EGFP mice, which constitutively express EGFP controlled by the CMV enhancer and the actin promoter, were used to trace periosteal and endosteal/marrow‐derived cells post P2 amputation. Mice were anesthetized using isoflurane as outlined above, and diaphyseal portions of the EGFP‐labeled P2 bone were harvested from unamputated hind limb digits two and four. The diaphyseal portion of the P2 bone was immediately grafted into newly fractured unlabeled NOD‐SCID mice hind limb P2 digits two and four, adjacent to the fracture gap. Several grafting designs were implemented to trace specific cellular components of the bone: (1) to trace both the periosteal cellular contribution as well as the endosteal/marrow contribution, we grafted the labeled bone with all components intact; (2) to trace solely the periosteal contribution post P2 amputation, we grafted P2 labeled bones with the endosteal/marrow tissues removed with a scalpel; (3) to trace solely the endosteal/marrow compartment post P2 amputation, we grafted P2 bones with the endosteal/marrow region intact, yet with the periosteum removed via stripping the tissue away with a scalpel; and (4) to test the cortical bone contribution post amputation, we removed the periosteum as well as the endosteal/marrow components of the labeled grafted bone. All samples were collected 11 days post grafting and fracture, when chondrogenesis was prevalent.

### Digit processing and histological staining

Digits were collected from mice at varying time points and fixed in buffered zinc formalin (Z‐Fix, Anatech Ltd, Battle Creek, MI) for 24−96 h at room temperature. Digits were decalcified using Decalcifier I (Surgipath, Leica Biosystems, Richmond, IL), a 10% formic acid solution, overnight to 24 h. Decalcified digits were processed through a graded ethanol series, xylenes, and immersed in paraffin wax. Digits embedded in paraffin wax were serial sectioned at 5 μm thickness. Prior to histological staining, slides were incubated at 60°C for 45 min, followed by incubation at 37°C for no less than 15 min, with subsequent deparaffinization with xylenes, a graded ethanol series, and eventual submersion in water. Mallory trichrome staining was performed to illustrate general histology. In order to visualize cartilage specifically, toluidine blue staining was performed. Slides were mounted using Permount Mounting Medium (Thermo Fisher Scientific, Waltham, MA). All slides were imaged using the Olympus BX60 microscope and DP72 camera, utilizing the DP2‐BSW software (Olympus America Inc., Center Valley, PA).

### Antibodies and immunohistochemistry

Digits were harvested and processed as described above in Digit Processing and Histological Staining. Antigen retrieval was performed using either proteinase K solution (Dako, Carpinteria, CA) and incubated at room temperature for 12 min, or heat retrieval performed in 1× citrate buffer solution (Dako). Slides were blocked using Protein Block (Dako) for 30 min to 1 h at room temperature. Incubation with primary antibody/antibodies was performed overnight at 4°C; they were washed in Tris buffered saline with Tween® 20 solution (Sigma‐Aldrich Co., St Louis, MO) and incubated in secondary antibody/antibodies for 45 min at room temperature. Slides were subsequently incubated in a phosphate buffered saline (Sigma‐Aldrich Co.) and DAPI (Invitrogen, Carlsbad, CA) solution, dried, and mounted with Prolong Gold (Invitrogen). Immunostaining for collagen II was carried out using the monoclonal mouse anti‐mouse collagen type II antibody (Acris Antibodies, San Diego, CA) and either the Alexa Fluor 488 or Alexa Fluor 647 goat anti‐mouse IgG secondary antibody (Invitrogen). Immunostaining for aggrecan was performed using the rabbit anti‐mouse aggrecan polyclonal antibody (EMD Millipore, Billerica, MA) and the Alexa Fluor 488 goat anti‐rabbit IgG secondary antibody (Invitrogen). Osteoblast immunostaining was performed using the rabbit anti‐osterix, SP7 polyclonal antibody (Abcam, Cambridge, UK) and the Alexa Fluor 488 goat anti‐rabbit IgG secondary antibody (Invitrogen). Immunostaining for GFP was carried out using the chick anti‐GFP polyclonal antibody (Novus Biologicals, Littleton, CO) and the Alexa Fluor 568 goat anti‐chick IgG secondary antibody (Invitrogen). All samples were imaged using the Olympus BX61 microscope, via the Slidebook software (Intelligent Imaging Innovations Inc., Denver, CO).

### Micro‐computed tomography (μCT) scans, volume measurements, and image processing

P2 digits were scanned from 1 DPA, fracture, or periosteum removal, and weekly thereafter for up to 12 weeks using the vivaCT 40 (SCANCO Medical, Wayne, PA) as described previously (Fernando et al. 2011).  Mice were anesthetized as outlined above.  Digits were scanned at 10.5 μm voxel size and energy of 45 kVp; 1000 projections per 180° were captured at 380 msec using continuous rotation.  Images were saved as a series of dicom files.  Image sequences were uploaded to ImageJ.  Using the BoneJ (Doube et al. 2010) (Version 1.2.1) Optimize Threshold Plugin for ImageJ, uploaded images were segmented. Three‐dimensional (3D) reconstructed images were created using the 3D viewer plugin, whereby bone volume changes were quantified directly from the 3D rendering using the BoneJ Volume Fraction Plugin.  Bone volume was normalized to total bone volume at 1 day post surgery. Changes in bone length were quantified from the 3D rendering using the ImageJ landmarks point tool. Bone length was normalized to length at 1 day post surgery.

## Supporting information

Additional Supporting Information may be found in the online version of this article at the publisher's website:

**Figure S1**. Bone length measurements over 28 days, normalized to 1 day post surgery, showing no change in bone length of the proximal fracture fragment and the amputated P2 bone from 1 day post surgery (*t* test, ±SEM, *P* < 0.05) and no statistical difference between the two groups at any time point (*t* test, ±SEM, *P* < 0.05).

**Figure S2**. (A) Tissue section of an intact GFP‐labeled P2 bone grafted into a NOD‐SCID fractured P2 digit, 11 DPF. No transgenic GFP fluorescence is observed associated with the grafted bone (outlined) after antigen retrieval. Red blood cells (RBCs) shown in yellow. (B) Immunostained sample of a periosteum and endosteum/marrow‐removed GFP‐labeled P2 bone grafted into a NOD‐SCID fractured P2 digit, 11 DPF. Inset of K′′; arrowheads indicate groups of red blood cells. Samples counterstained with DAPI. A, 50 μm; B, 20 μm.
