# Supplementary figures and images for: Analogous cellular contribution and healing mechanisms following digit amputation and phalangeal fracture in mice
Source: Regeneration (Oxf). 2016 Mar 9;3(1):39–51. doi: 10.1002/reg2.51 (PMC4857751; doi:10.1002/reg2.51)

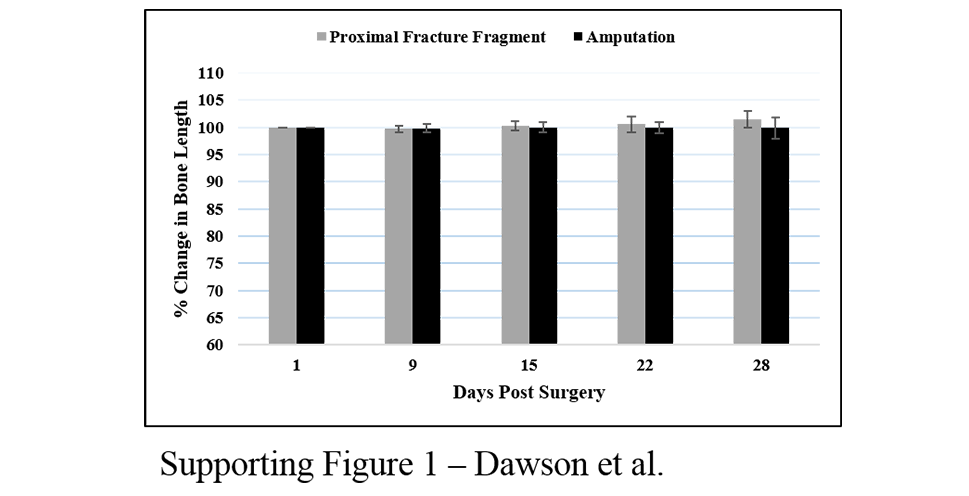

Supplement: Supplementary file 1 — Figure S1. Bone length measurements over 28 days, normalized to 1 day post surgery, showing no change in bone length of the proximal fracture fragment and the amputated P2 bone from 1 day post surgery (t test, ±SEM, P < 0.05) and no statistical difference between the two groups at any time point (t test, ±SEM, P < 0.05). [file REG2-3-39-s001.tif]

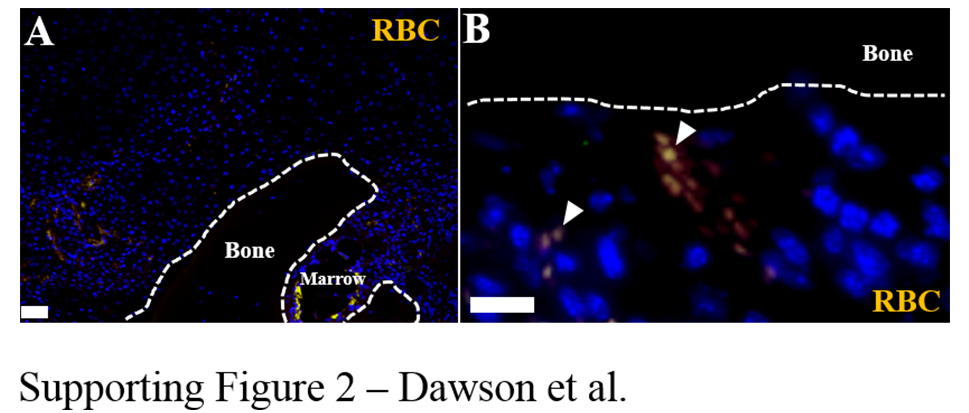

Supplement: Supplementary file 2 — Figure S2. (A) Tissue section of an intact GFP‐labeled P2 bone grafted into a NOD‐SCID fractured P2 digit, 11 DPF. No transgenic GFP fluorescence is observed associated with the grafted bone (outlined) after antigen retrieval. Red blood cells (RBCs) shown in yellow. (B) Immunostained sample of a periosteum and endosteum/marrow‐removed GFP‐labeled P2 bone grafted into a NOD‐SCID fractured P2 digit, 11 DPF. Inset of K′′; arrowheads indicate groups of red blood cells. Samples counterstained with DAPI. A, 50 μm; B, 20 μm. [file REG2-3-39-s002.tif]
